# In their own words: stressors facing medical students in the millennial generation

**DOI:** 10.1080/10872981.2018.1530558

**Published:** 2018-10-05

**Authors:** Monica R. Hill, Shelby Goicochea, Lisa J. Merlo

**Affiliations:** Department of Psychiatry, University of Florida College of Medicine, Gainesville, FL, USA

**Keywords:** Medical education, wellness, stressors, medical school, qualitative research

## Abstract

Background: Medical student exposure to stressors is associated with depression, burnout, somatic distress, decreases in empathy, serious thoughts about dropping out of medical school, suicidal ideation, and poor academic performance. Despite this, there have been no recent, multicenter, qualitative studies assessing medical students’ perceptions of their greatest stressor(s). Objective: The goal of this study was to identify the most significant stressors noted by medical students themselves, in order to inform the development of programs and policies to reduce medical student distress. Design: Medical students from the nine schools in the state of Florida were invited to complete an anonymous online questionnaire assessing wellness and distress. Students were notified that all responses were voluntary and that individual responses would not be linked to themselves or their program. This paper focuses on students’ responses to fixed-response items regarding their experience of stress and open-ended responses to the following question: ‘What do you consider to be the greatest stressor(s) facing medical students?’ Qualitative data were analyzed using the Grounded Theory method of data analysis. Results: Results confirmed the impact of several stressors highlighted in previous studies (e.g., excessive workload, difficulties with studying and time management, conflicts in work–life balance and relationships, medical school peer relations, health concerns, and financial stressors). However, students also reported unique system-level concerns that have not consistently been highlighted in past research (e.g., medical school administrative failures, concerns about lack of assistance with career planning, and assessment-related performance pressure. Conclusions: Though individually focused interventions have demonstrated some success, medical students self-report stressors that may be better addressed through system-level changes.

## Introduction

Medical students report higher levels of psychological distress than their same-age peers [1–6], despite having similar or healthier profiles than peers at the outset of medical school [7–9]. This suggests that medical education itself contributes to student distress. Studies have indeed documented that stress levels increase over the course of medical school, peaking either in the second year [7] or when students enter the medical wards [10].

While some stress may enhance academic performance [11], high levels of stress among medical students are associated with depression [7,12,13], burnout [14,15], and somatic complaints [13]. Anxiety and depression have been linked to medical students’ vulnerability to stress [16], and higher levels of psychological distress have been linked to decreases in empathy [17], serious thoughts about dropping out of medical school [5], suicidal ideation [5,10], and poor academic performance [18–20]. Previous research has explored student vulnerability to stress [4,21,22], and several studies have documented major stressors for medical students, including academics [15,18], lack of balance [23], relationships [18], poor student guidance/support [23], volume of information [18,23], finances [23], uncertainty of the future [15], lack of time to oneself [18], time and responsibility [23], and the need to succeed [18]. In addition, a recent review described six major themes associated with student distress: adjustment, ethical concerns, exposure to patient death and suffering, student mistreatment, personal life events, and educational debt [24].

However, these studies have relied on fixed-response items to assess student concerns. To our knowledge, there have been no recent, multicenter, qualitative studies designed to elicit directly from medical students what *they* consider to be their greatest stressor(s). An advantage of this methodology is that it allows students to freely list their concerns and thus provides a holistic picture of medical student perceptions regarding their stressors. Additionally, this methodology provides the opportunity for students to report stressors that may not have been adequately explored in quantitative studies. The present study used a mixed-methods approach to assess medical student perceptions of stressors impacting their current well-being.

## Methods

This project was part of an initiative to study medical student wellness in the state of Florida. The survey was created for this study by a panel of five epidemiological/clinical researchers who contributed to the initial pool of items, and content validity was established via consensus. The Florida medical school deans, administrators of the state physician health program, and medical students from multiple schools assessed face validity and provided feedback on items. No formal reliability analysis was conducted. The questionnaire included both quantitative and qualitative items assessing overall well-being, coping strategies, substance use, psychiatric symptoms, and suggestions for improving the medical school experience.

This study was exempted or approved by the IRBs at all participating medical schools and was supported by the Council of Florida Medical School Deans and the state physician health program. In the spring of 2014, student leaders at the nine medical schools in Florida invited all medical students to complete an anonymous questionnaire assessing medical student wellness and distress. Students were invited via e-mail and/or class announcements and provided a link to the survey, which was hosted on www.surveymonkey.com. Students were informed the survey would require approximately 30 min to complete and were not compensated for participation. They were assured that all responses were anonymous and could not be linked to individual students or their medical schools. This paper focuses on student responses to fixed-choice questions about medical school stressors, as well as the following open-ended question: ‘What do you consider to be the greatest stressor(s) facing medical students?’

### Data analysis

Quantitative data were analyzed using IBM SPSS v.24. Chi-square analyses were computed to explore differences by medical school class year and gender. Weighted averages were examined to determine the ranking of reported stress among the groups. Qualitative data were analyzed using the Grounded Theory method [25] of data analysis. Researchers (LJM and MRH) independently read student responses and generated a list of codes for the data set. The researchers then compared code lists and agreed upon a common set of codes. Student responses were coded by MRH using Atlas.ti and reviewed by LJM. Coding discrepancies were resolved through discussion. The coded data were organized by general themes, and representative student quotes were identified. Some quotes were edited slightly to enhance readability (i.e., typos were corrected).

## Results

### Quantitative results

Of the approximately 5000 medical students in Florida, 1137 students responded to the study survey. Because students were invited by student leaders at their school, it is unknown exactly how many students actually received the initial invitation and/or any reminders. A total of 987 students (87% of the study sample) completed the quantitative survey items related to stress during medical school and are included in the current analyses. The respondents (49.7% female, 37.4% male, 12.9% did not report gender) self-reported as 29.8% first year students, 25.7% second year students, 16.8% third year students, 14.8% fourth year students, 0.4% fifth year or higher students, and 12.5% did not report their class year).

Medical student respondents described their current stress as ‘severe and debilitating’ (11.2%), ‘significant but manageable’ (68.6%), ‘moderate’ (15.4%), ‘mild’ (3.8%), or ‘none’ (0.3%). They described the level of competitiveness among students in their medical school as ‘severe [hypercompetitive]’ (3.4%), ‘significant’ (23.4%), ‘moderate’ (37.0%), ‘mild’ (25.7%), or ‘none [very cooperative]’ (10.7%).

Students rated the degree to which various factors contribute to their stress. Table 1 shows these results, separated by class year in medical school. Academic workload and conflicts with work–life balance emerged as the top stressors across all years. However, there were significant differences between the classes in reported stress from academic workload (highest among first year students), competition with peers (highest among second year students), time spent commuting (highest in third year), conflicts in work–life balance (highest in third year), romantic relationship management (highest in third year), family demands (highest in third year), financial difficulties (lowest in first year), personal medical condition (highest in third year), and exposure to human suffering (highest in fourth year).

**Table 1.** Medical student perceptions of the degree to which various factors contribute to their stress by medical school class year.

Factors contributing to stress	Not at all (%)	A little (%)	A moderate amount (%)	A lot (%)	*X*^2^
Academic workload ● MS1 (*n* = 294) ● MS2 (*n* = 253) ● MS3 (*n* = 166) ● MS4 (*n* = 146) ● MS5+ (*n* = 4)	3.4 1.2 1.8 2.1 0.0	7.5 10.7 9.6 17.8 0.0	40.1 36.4 36.7 47.9 75.0	52.0 51.8 51.8 32.2 25.0	28.8**
Inadequate study habits● MS1 (*n* = 294) ● MS2 (*n* = 253) ● MS3 (*n* = 166) ● MS4 (*n* = 146) ● MS5+ (*n* = 4)	19.0 23.7 25.3 31.5 0.0	43.5 40.3 33.1 34.2 25.0	21.8 24.1 24.1 22.6 25.0	15.6 11.9 17.5 11.6 50.0	19.0
Poor time-management skills● MS1 (*n* = 294) ● MS2 (*n* = 252) ● MS3 (*n* = 166) ● MS4 (*n* = 146) ● MS5+ (*n* = 4)	26.2 28.6 27.7 37.0 0.0	43.9 40.5 38.6 36.3 75.0	19.4 22.2 20.5 18.5 25.0	10.5 8.7 13.3 8.2 0.0	12.0
Competition with peers● MS1 (*n* = 294) ● MS2 (*n* = 253) ● MS3 (*n* = 165) ● MS4 (*n* = 146) ● MS5+ (*n* = 4)	40.6 32.8 21.2 39.0 25.0	38.9 40.7 45.5 39.9 25.0	16.0 16.6 24.2 13.0 25.0	4.4 19.9 9.1 8.2 25.0	28.6**
Time spent commuting● MS1 (*n* = 294) ● MS2 (*n* = 253) ● MS3 (*n* = 166) ● MS4 (*n* = 146) ● MS5+ (*n* = 4)	60.9 59.3 37.3 57.5 75.0	30.6 32.0 36.1 27.4 25.0	6.5 5.9 18.1 10.3 0.0	2.0 2.8 8.4 4.8 0.0	47.2***
Conflicts in work–life balance● MS1 (*n* = 294) ● MS2 (*n* = 253) ● MS3 (*n* = 166) ● MS4 (*n* = 145) ● MS5+ (*n* = 4)	29.6 22.9 10.8 19.3 0.0	26.9 32.0 28.3 30.3 25.0	30.6 26.5 31.9 34.5 50.0	12.9 18.6 28.9 15.9 25.0	38.0***
Romantic relationship management● MS1 (*n* = 294) ● MS2 (*n* = 253) ● MS3 (*n* = 166) ● MS4 (*n* = 146) ● MS5+ (*n* = 4)	31.3 34.0 18.7 27.4 0.0	34.7 31.6 31.3 29.5 50.0	22.4 22.1 27.7 26.0 50.0	11.6 13.8 22.3 17.1 0.0	22.7*
Family demands● MS1 (*n* = 294) ● MS2 (*n* = 253) ● MS3 (*n* = 166) ● MS4 (*n* = 146) ● MS5+ (*n* = 4)	47.6 40.7 27.1 34.2 75.0	33.0 35.2 38.6 34.9 0.0	13.3 14.6 19.3 20.5 25.0	6.1 9.5 15.1 10.3 0.0	30.3**
Financial difficulties● MS1 (*n* = 294) ● MS2 (*n* = 253) ● MS3 (*n* = 164) ● MS4 (*n* = 146) ● MS5+ (*n* = 4)	48.1 39.5 32.3 28.8 25.0	31.3 30.8 38.4 39.7 50.0	15.4 17.0 15.2 19.9 25.0	5.1 12.6 14.0 11.6 0.0	29.8**
Psychological/Psychiatric condition● MS1 (*n* = 294) ● MS2 (*n* = 253) ● MS3 (*n* = 166) ● MS4 (*n* = 146) ● MS5+ (*n* = 4)	66.7 60.9 59.6 61.0 25.0	20.7 21.3 18.1 21.9 25.0	8.8 9.1 13.3 10.3 25.0	3.7 8.7 9.0 6.8 25.0	14.6
Other medical condition● MS1 (*n* = 294) ● MS2 (*n* = 253) ● MS3 (*n* = 163) ● MS4 (*n* = 145) ● MS5+ (*n* = 4)	81.6 74.7 73.0 76.6 50.0	11.9 19.0 15.3 16.6 25.0	4.4 3.2 9.2 4.8 0.0	2.0 3.2 2.5 2.1 25.0	23.0*
Exposure to human suffering● MS1 (*n* = 291) ● MS2 (*n* = 248) ● MS3 (*n* = 162) ● MS4 (*n* = 144) ● MS5+ (*n* = 4)	70.1 72.2 48.8 45.1 100.0	24.4 20.6 40.1 38.9 0.0	4.5 3.6 6.2 15.3 0.0	1.0 3.6 4.9 2.8 0.0	68.1***

Notes: MS1: First year medical student; MS2: second year medical student; MS3: third year medical student; MS4: fourth year medical student; MS5+: fifth year student or higher. *p < 0.05; **p < 0.01; ***p < 0.001.

When examining the impact of various stressors by gender, few differences emerged. However, as seen in Table 2, there were gender differences related to academic workload, poor time-management skills, personal medical conditions, and exposure to human suffering, with female students reporting greater stress in each area. Total ratings for all study participants, including those who elected not to report medical school class year and/or gender, are also included in Table 2.

**Table 2.** Medical student perceptions of the degree to which various factors contribute to their stress by gender.

Factors contributing to stress	Not at all (%)	A little (%)	A moderate amount (%)	A lot (%)	*X*^2^
Academic workload● Male (*n* = 369) ● Female (*n* = 490) ● Total (*n* = 985)	2.2 0.4 1.3	13.6 8.4 10.6	38.2 41.0 39.5	46.1 50.2 48.6	12.1**
Inadequate study habits● Male (*n* = 369) ● Female (*n* = 490) ● Total (*n* = 985)	26.6 21.2 22.6	37.7 40.2 39.7	20.1 25.1 24.0	15.7 13.5 13.7	6.0
Poor time-management skills● Male (*n* = 369) ● Female (*n* = 489) ● Total (*n* = 984)	32.0 26.4 28.5	37.4 57.2 40.7	18.4 21.9 21.1	12.2 8.6 9.8	7.9*
Competition with peers● Male (*n* = 369) ● Female (*n* = 490) ● Total (*n* = 983)	34.0 34.4 33.8	44.0 38.4 40.6	14.9 19.0 18.1	7.1 8.2 7.5	4.0
Time spent commuting● Male (*n* = 369) ● Female (*n* = 490) ● Total (*n* = 985)	55.8 55.3 54.2	31.7 31.2 32.1	8.4 9.6 9.4	4.1 3.9 4.3	0.4
Conflicts in work–life balance● Male (*n* = 369) ● Female (*n* = 489) ● Total (*n* = 983)	22.5 21.7 22.8	29.3 29.2 29.4	29.5 31.3 30.7	18.7 17.8 17.1	0.4
Romantic relationship management● Male (*n* = 369) ● Female (*n* = 490) ● Total (*n* = 985)	26.3 30.4 28.5	31.2 33.3 32.3	25.2 23.3 24.2	17.3 13.3 15.0	4.1
Family demands● Male (*n* = 369) ● Female (*n* = 490) ● Total (*n* = 985)	42.8 36.9 39.9	32.2 36.9 34.7	14.6 17.1 16.0	10.3 9.0 9.3	4.4
Financial difficulties● Male (*n* = 366) ● Female (*n* = 490) ● Total (*n* = 981)	41.3 37.8 39.8	33.3 34.3 33.4	17.2 16.3 16.4	8.2 11.6 10.4	3.2
Psychological/Psychiatric condition● Male (*n* = 369) ● Female (*n* = 490) ● Total (*n* = 985)	67.5 58.8 62.4	18.2 22.4 21.4	8.7 11.0 9.5	5.7 7.8 6.6	6.9
Other medical condition● Male (*n* = 366) ● Female (*n* = 489) ● Total (*n* = 981)	83.6 72.0 76.0	10.9 18.8 16.0	3.3 6.3 5.5	2.2 2.9 2.4	16.4***
Exposure to human suffering● Male (*n* = 360) ● Female (*n* = 485) ● Total (*n* = 970)	67.8 58.4 62.9	25.8 30.3 28.1	4.7 7.6 6.4	1.7 3.7 2.6	10.2*

Notes: ‘Total’ responses exceed the number of male and female responses because some students elected not to report their gender. Chi-square analyses compare male and female responses. *p < 0.05; ** = *p* < 0.01; ***p < 0.001.

**Table 3.** Greatest stressors facing medical students based on responses to an open-ended question.

**Theme**	**Number** **of students who mentioned the theme in their response**
Medical school workload	333
Performance pressure	306
Medical school structure, administration, and faculty	73
Time constraints and lack of balance	285
Peer relations and social environment	102
Negative health impact of school	38
Career planning and concerns about the future	93
Financial concerns	147

Student self-reported stress was also significantly associated with other measures of mental health. Those who described ‘severe and debilitating’ stress were more likely to report being unhappy (particularly moderately or extremely unhappy) than their peers (*X*^2^ = 283.18, *p* < .001). Those with either ‘significant but manageable’ or ‘severe and debilitating’ stress were more likely to report being less happy than they were before medical school (*X*^2^ = 250.03, *p* < .001), and more likely to self-report a depressive episode (*X*^2^ = 146.41, *p* < .001), or symptoms of generalized anxiety (*X*^2^ = 163.95, *p* < .001), social anxiety (*X*^2^ = 62.01, *p* < .001), obsessive-compulsive (*X*^2^ = 37.87, *p* < .001), ADHD (*X*^2^ = 55.44, *p* < .001), or sleep disorder (*X*^2^ = 47.12, *p* < .001) that began or worsened during medical school. Self-reported stress was not significantly associated with self-reported eating disorder (*X*^2^ = 19.08, ns) or gambling disorder symptoms (*X*^2^ = 1.88, ns).

With regard to coping strategies, there were several significant differences among students. Those who reported lower stress reported more frequently: exercising (*X*^2^ = 32.61, *p* = 0.001), playing sports (*X*^2^ = 49.17, *p* < 0.001), reading (*X*^2^ = 45.70, *p* < 0.001), participating in extracurricular activities (*X*^2^ = 39.64, *p* < 0.001), socializing without alcohol (*X*^2^ = 32.84, *p* < 0.001), and participating in hobbies (*X*^2^ = 34.29, *p* = 0.001). There were no differences in using sleep, listening to music, social activities involving alcohol, playing with pets, cooking, or talking to a confidant as a coping strategy. Watching television, eating, and skipping lectures had mixed results, with students reporting both very high and very low stress engaging in these activities as a method of coping. Finally, some activities were fairly uncommon across all groups, including yoga/pilates, meditation, relaxation training, prayer/worship, playing video games, playing music, massage/spa services, shopping, drinking alone, using mood-altering drugs, and taking ‘fun’ classes.

### Qualitative results

Of the 987 study participants, 864 responded to the open-ended question assessing ‘greatest stressor(s)’ faced by medical students. Twelve students typed ‘N/A,’ ‘X,’ or a similar response, resulting in a total of 852 responses that were appropriate for analysis. Table 3 lists the distribution of responses per theme. Many students provided responses that included multiple themes. Representative quotes for each theme are listed below.

#### Medical school workload

Academic workload was most frequently noted as a stressor, with 333 students referring to this theme (e.g., ‘I NEVER have the feeling of being ‘done’ for the day, or even for the week. There is always more that needs to be done’). Specifically, students cited concerns about the overwhelming volume of detailed information they were expected to master (‘[I feel stressed by] the amount of material needed to learn … the expectation to master it and know it and be able to recall it on demand’) and the pace of the curriculum (‘School places too much on us without hours in the day’). Students also noted frustration regarding ‘unnecessary’ academic requirements (‘[I feel stressed by] the overwhelming amount of extra work that doesn’t impact our careers as doctors’) and described expectations placed on them as unrealistic (‘[We must] study and know everything, do research, participate in extracurricular, volunteer, sacrifice personal time and interests, know how to deal with major illness and their families’).

#### Performance pressure

In this sample, 306 students described pressure to perform as a significant stressor. Students noted both internal pressure they put on themselves (‘[I feel] stress to succeed due to fear of failure’) and external pressure to perform academically (‘[There is] performance pressure from myself, parents, professors/attendings’). Indeed, many students, particularly those at schools with a ranked/graded system, noted significant pressure to perform academically (‘At my school, all students are obsessed with their quartiles in the class, determined solely by grades’). This was compounded by perceived unfairness in grading (‘subjectiveness of clinical year grades’) and relentless assessment (‘being constantly evaluated … constantly, this is ridiculous, and is not compatible with enjoying day to day work’). They noted reasons for concern about their performance (‘[I’m] striving to get the best grades and best USMLE scores in order to be a competitive applicant [for residency]’) and described significant anxiety related to high-stakes board examinations (‘In the first two years, the only thing that matters is Step 1. It’s always on your mind’). Finally, students described difficulty adjusting to the medical school curriculum, resulting in worse performance than expected (‘The frustration comes when previously trusted study habits fail, and new study strategies are difficult to adapt to and are of unknown usefulness until they have been tried’). Students noted that this further contributes to their distress (‘The [medical school] process breeds self-doubt and breaks your confidence’).

#### Medical school structure, administration, and faculty

A total of 73 students expressed significant frustration with the structure and/or employees of their medical schools. They described a perception of the medical school culture as unfriendly to students (‘[I experience] general antipathy of school and attending physicians towards students’), as well as feeling unable to receive the support they need (‘[There is a] lack of adequate and helpful resources for support [mental health, conflict resolution, tutors]’). They cited communication problems (‘Communication amongst the administration, professors, and students has had some major lapses that have been worsened by technical malfunctions’), coupled with confusion about academic requirements (‘I’ve often felt the hardest part about school is knowing where they want us when and what is due when’). Further, scheduling concerns were noted by a number of medical students; in particular, due to inefficient use of time (‘Medical school [is] not efficiently using time to teach all the material that is necessary to be successful during 3rd year rotations’) and unpredictability (‘The biggest preventable stressor at my school is scheduling. We don’t have a set schedule, we are almost always there for most business hours M-F, they will regularly schedule mandatory activities at the last minute, and they don’t give us our schedule very far in advance’). Students described poorly organized course material as an additional logistical stressor (‘misdirection and organizational issues of the class material delivered to us’), as well as concerns about the qualifications of certain faculty (‘[We are] presented with information by individuals not trained to teach and having to learn the material by ourselves’). Indeed, several students reported being concerned about poor curricular preparation (‘We have to continuously study for USMLE exams on our own because they don’t match up with the school’s normal coursework’).

#### Time constraints and lack of balance

Concerns about time commitments and work–life balance were noted by 285 students. They reported difficulties with time management (‘[I feel stressed by] poor study/time management skills and schools that don’t effectively address them’) and insufficient time to complete required tasks (‘It feels like most days we barely have time to breathe let alone study and do everything else’). Students in the preclinical years expressed concern about giving up time they felt could be better spent on other activities (‘The mandatory activities are perfunctory, and waste precious time’), whereas those in the clinical years expressed concerns about competing responsibilities (‘[Trying to manage] work hours during certain clerkships; i.e. 80 h during surgery, and figuring out when to study’). They lamented a lack of personal time (‘You sometimes lose touch with yourself and those you love, which can be very difficult’), noting that they tended to prioritize school when time was short (‘[There is a] lack of time to invest in a well-rounded lifestyle. Non-school activities can easily be sacrificed’). Students reported being cognizant of the lack of balance in their lives (‘It is difficult to maintain “balance” even though that is drilled into us’), noting that this seemed to be implicitly encouraged/expected (‘[The] culture among doctors [is] that ambition is more important than balance’). Accordingly, many students reported that their relationships had suffered (‘I also don’t have time for much outside of medical school, so that puts stress on my relationships outside of school’). For others, medical school had caused them to miss major family events or ‘put off life events (i.e., getting married, having kids) until after school.’

#### Peer relations and social environment

Concerns about the negative impact of peer interactions were noted by 102 students, with 74 students commenting specifically on the competitive environment within their medical school (‘[There is] competition among other students, students cheating, bullying, abusing stimulant medication, that places honest students at a disadvantage’). They further suggested that the competition between students compromised professional values (‘It seems that we [have] all of this pressure to be the best and out-do the medical student that is next to us that we forget why we joined this practice in the first place’). Additionally, students noted social conflicts within the medical school community (‘social stresses with classmates, residents, attending[s], etc.’) and indicated that peer interactions sometimes increased distress (‘students transfer their anxieties to each other’).

#### Negative health impact

Decreased participation in healthy activities and/or increased health problems were noted by 38 students, with lack of sleep in medical school emerging as the primary health concern (‘Sleep deprivation is pervasive among medical students and seems to be the norm’). The students also described difficulty prioritizing activities to maintain a healthy lifestyle (‘It can be difficult to make or find time to cook healthfully, exercise regularly, sleep enough, live well. In particular, it’s difficult to schedule doctor’s appointments to keep yourself well and healthy’). Some students noted a decline in their coping ability (‘A lot of students turn to drinking for relaxation. My friends drink significantly more than they did at the beginning of medical school’) with limited options for improving their psychological functioning (‘[We have] no access to mental health professionals’).

#### Career planning and concerns about the future

Students (*n* = 93) also described anxiety related to their training, career plans, and the future of medicine. The gap in available residency training slots stoked fears (‘[There is a] decreasing number of residency positions and increasing numbers of medical graduates’), particularly among students who felt underinformed about the application process (‘[I have a] lack of real knowledge about what matters when applying for residency’). Other students described concerns about their future practice as a physician due to recent changes in the US health-care system (‘[I worry about] the unknown future of the medical profession and practice [i.e., Obamacare]’) and the business-side of medicine (‘the hours, paperwork, litigation, debt, and lack of financial reimbursement’). Indeed, some students expressed worry about their ability to commit to a specialty (‘[I stress about] deciding which field I want to go into for the rest of my life’) and questioned the likelihood of achieving career satisfaction (‘uncertainty of whether we will be able to get a satisfying job when we finish’). Finally, some students noted discomfort with the significance and responsibility of being a physician (e.g., ‘[It is stressful] realizing you are expected to know a ton of stuff that will potentially save someone’s life’).

#### Financial concerns

Finally, 147 students noted financial concerns. These students expressed frustration with their current lack of money (‘Being surrounded by your peers [who aren’t in medical school] who have jobs and paychecks and weekends [when I have] no job, huge debt, and no weekends’) and inability to purchase basic supplies (‘It’s scary to think that I may not have enough loan money to cover my basic needs through summer without having to take out another loan that charges higher interest’). Indeed, many mentioned loan burden (‘DEBT!!!’) coupled with fears about their future earning potential (‘[I worry about] health care reimbursement and if I will be compensated for all of the sacrifices I’ve made thus far/[be] able to pay off loans’). Some students even reported concerns that they would be unable ‘to pursue desired specialties due to inadequate reimbursement of those specialties compared to student loan debt.’

## Discussion

Comparison of quantitative and qualitative data demonstrated some overlap regarding student perceptions of the stressors they experience during medical school. Excessive workload, difficulties with studying and time management, conflicts in work–life balance and relationships, medical school peer relations, health concerns, and financial stressors were noted as significant issues, both in a fixed-response item near the beginning of the study questionnaire and in an open-response item near the end. These results were fairly consistent with other studies [15,18,23,26]. However, Dyrbye and colleagues previously described six overarching causes of student distress [24], and three of these were not widely endorsed by students on the fixed-response items in the present study: ethical concerns, human death and suffering, and personal life events. Furthermore, student responses to the open-ended item highlighted some issues that have not previously received significant attention. Specifically, students noted medical school administrative failures (e.g., unsupportive learning environment, inefficient scheduling, communication problems), concerns about career planning (e.g., fears about residency placement, choosing a specialty, and the future of the health-care system; lack of confidence in physician role), and performance pressure (e.g., constant assessment, subjective grading, and high-stakes examinations) as significant stressors. Though the impact of these stressors likely varies between schools, based on their policies and curricular structure, it is apparent that more work is needed to assess their contribution to medical student distress and burnout.

The present study also provides some insight into differences in challenges faced by students in the preclinical years (i.e., first 2 years of medical school) compared to the clinical years (i.e., the third and fourth years). With the exception of time management challenges and deficiencies in study skills, each stressor had varying impact across the four class years. The most significant stressor in the first year appears to be related to the transition from college to medical school (i.e., increased workload), which the students noted as contributing to their first experience ‘not being the smartest’ in their class. Then, in second year, the stress associated with a more competitive/less supportive school environment seems to peak. The third year students appear to struggle most with balancing school/clinical work with their other life responsibilities, whereas the fourth year students experience the most direct confrontation with mortality and suffering. Such data may be useful to inform the development of longitudinal wellness programs for medical students, in order to best meet the needs of students in each year of school.

Finally, the observed gender differences in reported impact of various stressors complement previous research examining gender differences in physician burnout and professional satisfaction. For example, in the present study, female students endorsed higher distress from their exposure to human suffering than male students. This is consistent with previous research demonstrating that female physicians are more likely to experience emotional exhaustion at the onset of burnout, compared to male physicians who experience depersonalization as the initial symptom [27]. Similarly, female students in the present study reported more difficulty managing their academic workload and more problems with time management. This may relate to the fact that women tend to have additional domestic and parenting responsibilities compared to their male counterparts. Research has demonstrated this to be the case for female versus male physicians [28], but the present data suggest that the differences may be apparent by the time of medical school. As a result, female medical students, in particular, may benefit from learning strategies to share household tasks more equitably with their partner and/or utilize organizational and time management strategies more effectively. Future research should explore the benefit of such interventions.

Indeed, the current results support the need for additional wellness interventions for medical students. Research has demonstrated some benefit to such programs in terms of mitigating medical student stress and distress [29,30]. Similarly, system-level interventions such as changing to a pass/fail grading system [26,31–33], curricular restructuring [26,34], and introduction of mindfulness training [35–40] have demonstrated improvements in student well-being. Each of these interventions appears to directly target some of the primary stressors noted by students in the present study (e.g., decreasing workload and academic pressure, limiting competition and promoting cooperation among students, teaching skills to enhance mental health), which may explain the positive results. However, the present results suggest that other system-based interventions should also be considered. For example, students reported a desire for more efficient and consistent scheduling of classes; better communication between administration, faculty, and students; increased mentoring and career planning services; improved access to mental health professionals; and assistance with financial concerns.

The potential impact of introducing such changes has been exhibited by the experiences of two innovative medical school programs. Vanderbilt University School of Medicine introduced a comprehensive medical student wellness initiative that focused on improving mentoring and counseling access, increased social interaction amongst medical students, teaching skills to improve physical and mental health (e.g., healthy cooking, group exercise, mindfulness training), and devoting curricular time to ‘personal development of physicians-in-training’ [41]. Participation in the program elements was substantial, and it received positive feedback from students. Similarly, St. Louis University School of Medicine implemented multifaceted curricular reforms that resulted in reduced medical student anxiety, stress, and depressive symptoms. Their program included reduction in the level of detail covered in courses, student-centered scheduling changes, decreased course contact hours, introduction of collaborative ‘learning communities,’ switch to pass/fail grading, and introduction of resilience and mindfulness training [26]. This program was well received by students, who noted higher levels of satisfaction with the medical school wellness program throughout its implementation, and was associated with improvements in average Step 1 scores. Together with results of the present study, these findings support the widespread adoption of system-based reforms to address the growing problem of medical student distress.

### Limitations

Although the present sample was relatively large and included students from a variety of medical school settings (i.e., allopathic and osteopathic, public and private, traditional and problem-based learning curricula, pass–fail and graded systems), it is unclear whether response bias affected the results by excluding responses of students who chose not to participate. Additionally, due to the anonymity of the study, it was not possible to explore whether specific school characteristics (e.g., type of curriculum, grading system, and size of student body) affected students’ perceptions of their stressors.

### Conclusions and future directions

Medical student wellness is a significant concern that has recently received increased attention. Though individually focused interventions have demonstrated some success, medical students self-report stressors that may be better addressed through system-level changes. For example, future research should explore the impact of interventions that focus on implementing efficient schedules with built-in flexibility for students to address personal/family needs, providing students with increased financial planning and budgeting assistance, promoting clear expectations for students coupled with regular, open, and accessible communication with the administration and faculty, and improving access to faculty mentors and professional role models to assist with career planning and work–life balance.

## Supplementary Material

Supplemental Material
