# Supplementary material for: In their own words: stressors facing medical students in the millennial generation
Source: Med Educ Online. 2018 Oct 5;23(1):1530558. doi: 10.1080/10872981.2018.1530558 (PMC6179084; doi:10.1080/10872981.2018.1530558)
Supplement: Supplemental Material [file ZMEO_A_1530558_SM8845.pdf]

Florida Medical Student Wellness Survey

Thank you for participating in this important survey of medical student wellness, which is being conducted at all medical schools in Florida. **Your responses cannot be linked to you in any way.** Therefore, you can be completely candid when responding.

This project represents a major priority in medical school training. We want to identify areas where support could be provided to help medical students succeed.

Due to the importance of the study, **all data will remain anonymous**, and results will be reported in aggregate form (meaning, it will even be impossible to determine which school students attend). No email addresses or IP addresses are being collected.

The following questionnaire asks a variety of questions about medical student wellness, including personal health, stressors, coping strategies, risky behaviors, resources, quality of life, etc. Some of the items are personal/sensitive in nature. Please remember that all responses will be kept completely anonymous. You may choose to skip any items you do not wish to answer, or discontinue the survey at any time.

This survey has been reviewed and approved by the appropriate IRBs, and each agreed there were no significant risks to your participation. There are no direct benefits to you for participating in the study, though results may be used to improve the medical school experience in the future. If you have any questions or concerns about your participation in this study, please contact the Principal Investigator, Dr. Lisa Merlo, at 352-265-9624 or lmerlo@ufl.edu

The questionnaire should take 15-30 minutes to complete. Thank you for answering all of the questions, and for doing so honestly.

1. In order to participate, please click on the button below to confirm that you understand this survey is completely anonymous and that your answers cannot be linked to you in any way.

- ○ I understand that my responses are anonymous and wish to participate

THESE FIRST ITEMS ASK ABOUT YOUR OVERALL WELL-BEING AS A MEDICAL STUDENT.

2. Overall, how would you rate your PHYSICAL health during the past 4 weeks?

- ○ Excellent
- ○ Very Good
- ○ Good
- ○ Fair
- ○ Poor
- ○ Very Poor

3. Overall, how would you rate your physical health before beginning medical school?

- ○ Excellent
- ○ Very Good
- ○ Good
- ○ Fair
- ○ Poor
- ○ Very Poor

4. Since beginning medical school, how has your general physical health changed?

- Significantly Worse
- Moderately Worse
- A Little Worse
- No Change
- A Little Improved
- Moderately Improved
- Significantly Improved

5. Overall, how would you rate your PSYCHOLOGICAL health during the past 4 weeks?

- Excellent
- Very Good
- Good
- Fair
- Poor
- Very Poor

6. Overall, how would you rate your psychological health before beginning medical school?

- Excellent
- Very Good
- Good
- Fair
- Poor
- Very Poor

7. Since beginning medical school, how has your general psychological health changed?

- Significantly Worse
- Moderately Worse
- A Little Worse
- No Change
- A Little Improved
- Moderately Improved
- Significantly Improved

8. During the past 4 weeks, how much energy did you have?

- Very Much
- Quite a Lot
- Some
- A Little
- None

9. In the past 4 weeks, how much sleep have you gotten each night, on average?

- No sleep
- 1 hour
- 2 hours
- 3 hours
- 4 hours
- 5 hours
- 6 hours
- 7 hours
- 8 hours
- 9 hours
- 10 hours
- 11 hours
- 12 or more hours

10. Which do you consider this to be?

- Significantly less sleep than needed
- Slightly less sleep than needed
- Just the right amount of sleep
- Slightly more sleep than needed
- Significantly more sleep than needed

Florida Medical Student Wellness Survey

11. In the past 4 weeks, how would you describe your eating habits?

	Very healthy	Fairly healthy	Fairly unhealthy	Very unhealthy
Amount of food consumed	◯	◯	◯	◯
Quality of food consumed	◯	◯	◯	◯
Amount of sugary beverages consumed	◯	◯	◯	◯
Amount of caffeine consumed	◯	◯	◯	◯

THE NEXT ITEMS ASK ABOUT YOUR MEDICAL SCHOOL TRAINING EXPERIENCE, INCLUDING PROBLEMS THAT HAVE BEEN REPORTED BY OTHER MEDICAL STUDENTS. WE WILL NEVER ASK FOR ANY INDENTIFYING INFORMATION, INCLUDING THE NAME OF YOUR MEDICAL SCHOOL, SO YOU CAN ANSWER HONESTLY.

12. Currently, how satisfied are you with your medical school training experiences?

- ◯ Very Satisfied
- ◯ Somewhat Satisfied
- ◯ Indifferent
- ◯ Somewhat Dissatisfied
- ◯ Very Dissatisfied

13. How concerned are you about:

	Not at all concerned	A little concerned	Moderately concerned	Very concerned	Extremely concerned
Your ability to pay bills currently?	◯	◯	◯	◯	◯
Your student loan debt?	◯	◯	◯	◯	◯
Your financial future as a physician?	◯	◯	◯	◯	◯

14. During your medical school training, have you been negatively affected by someone else's

	Yes	No
psychiatric or emotional distress?	◯	◯
disruptive behavior?	◯	◯
substance use?	◯	◯
sexual harassment?	◯	◯
discrimination towards you?	◯	◯
violent behavior?	◯	◯
bullying behavior?	◯	◯
other problem?	◯	◯

(please specify)

15. During your medical training, has anyone in your medical school ever expressed concern that YOU exhibit

	Yes	No
psychiatric or emotional distress?	◯	◯
disruptive behavior?	◯	◯
substance use?	◯	◯
sexual harassment of others?	◯	◯
discrimination towards others?	◯	◯
violent behavior?	◯	◯
bullying behavior?	◯	◯
other problem?	◯	◯

(please specify)

16. Bullying in medical school may include things like name-calling, humiliating students in front of peers, threatening students with bad grades or a ruined career, or even pushing a student or throwing a medical instrument at him or her. To be considered bullying, it would be in excess of what you feel is reasonable for students who are "paying their dues" as part of training.

Have you ever been subjected to any of the following in your medical school training?

	Yes	No
bullying from an attending physician?	◯	◯
bullying from a classroom professor?	◯	◯
bullying from a resident or fellow?	◯	◯
bullying from another medical student?	◯	◯
bullying from medical school administration?	◯	◯
bullying from a nurse?	◯	◯
bullying from other staff?	◯	◯

17. Have you ever observed another student being subjected to any of the following in your medical school training?

	Yes	No
bullying from an attending physician?	◯	◯
bullying from a classroom professor?	◯	◯
bullying from a resident or fellow?	◯	◯
bullying from another medical student?	◯	◯
bullying from medical school administration?	◯	◯
bullying from a nurse?	◯	◯
bullying from other staff?	◯	◯

18. How would you describe the level of competitiveness among students in your medical school?

- ◯ Severe (hypercompetitive)
- ◯ Significant
- ◯ Moderate
- ◯ Mild
- ◯ None (very cooperative)

19. How would you describe the level of stress you have experienced as a medical student?

- ◯ Severe, and debilitating
- ◯ Significant, but manageable
- ◯ Moderate
- ◯ Mild
- ◯ None

20. How much do the following factors contribute to your level of stress?

	Not at all	A little	A moderate amount	A lot
Academic workload	○	○	○	○
Inadequate study habits	○	○	○	○
Poor time-management skills	○	○	○	○
Competition with peers	○	○	○	○
Time spent commuting	○	○	○	○
Conflicts in work-life balance	○	○	○	○
Romantic relationship management	○	○	○	○
Family demands	○	○	○	○
Financial difficulties	○	○	○	○
Psychological/psychiatric condition	○	○	○	○
Other medical condition	○	○	○	○
Exposure to human suffering	○	○	○	○

Other (please specify)

[ ]

21. Have you ever believed it would be better if you were dead?

- ○ Yes, both before and during medical school
- ○ Yes, only during medical school
- ○ Yes, only before medical school
- ○ No, never

22. Have you ever thought of committing suicide?

- ○ Yes, both before and during medical school
- ○ Yes, only during medical school
- ○ Yes, only before medical school
- ○ No, never

23. Listed below are National Suicide Hotlines, which provide free counseling:
1-800-SUICIDE (1-800-784-2433)
1-800-273-TALK (1-800-273-8255)

Please click "Next" to continue

○ Next

NEXT WE ASK ABOUT VARIOUS STRATEGIES THAT INDIVIDUALS USE TO COPE WITH PHYSICAL PROBLEMS, PSYCHOLOGICAL SYMPTOMS, AND STRESS. PLEASE ANSWER AS HONESTLY AS POSSIBLE.

24. During medical school, how often have you used these methods of stress reduction?

	Frequently	Occasionally	Rarely	Never
Exercise	○	○	○	○
Sports	○	○	○	○
Yoga/Pilates	○	○	○	○
Meditation	○	○	○	○
Relaxation training	○	○	○	○
Prayer or worship	○	○	○	○
Television/movies	○	○	○	○
Video games	○	○	○	○
Sleeping	○	○	○	○
Skipping lectures	○	○	○	○
Playing music	○	○	○	○
Listening to music	○	○	○	○
Massage or other spa services	○	○	○	○
Reading	○	○	○	○
Extracurricular activities	○	○	○	○
Talking to confidant	○	○	○	○
Shopping	○	○	○	○
Eating	○	○	○	○
Social activities without alcohol	○	○	○	○
Social activities involving alcohol consumption	○	○	○	○
Drinking alcohol alone	○	○	○	○
Using mood-altering drugs	○	○	○	○
Hobbies	○	○	○	○
Playing with pet(s)	○	○	○	○
Cooking	○	○	○	○
	○	○	○	○

Taking "fun" classes	◯	◯	◯	◯
Other	◯	◯	◯	◯

(please specify)

25. During medical school, have you ever felt you could benefit from resources to help students who are struggling emotionally or psychologically?

- ◯ Yes, often
- ◯ Yes, once in a while
- ◯ No, never

26. Are you aware of the following resources for medical students who are struggling emotionally or psychologically:

	No	Yes
Counseling/wellness center within the medical school?	◯	◯
Counseling/wellness center within the university?	◯	◯
Private mental health services in the community?	◯	◯
Professionals Resource Network?	◯	◯

27. Which of the following resources have you utilized during medical school? (select all that apply)

- ☐ None
- ☐ Counseling/wellness center within the medical school
- ☐ Counseling/wellness center within the university
- ☐ Private mental health services in the community
- ☐ Professionals Resource Network
- ☐ Other

(please specify)

28. Please comment on how useful/helpful you found the resource(s) to be.

29. To what extent would the following potential barriers impact your likelihood of seeking help from the resources listed above?

	Significant Barrier	Minimal Barrier	No Barrier
Concerns about confidentiality	○	○	○
Concerns about negative impact on academic career	○	○	○
Concerns about negative impact on licensure	○	○	○
Concerns about negative impact on professional career	○	○	○
Cost	○	○	○
Difficulty scheduling around other obligations	○	○	○
Inconvenient location	○	○	○
Lack of time	○	○	○
Unaware of how to access help	○	○	○
Other	○	○	○

(please specify)

30. Has anyone ever discouraged you from seeking support for psychological or emotional difficulties due to concerns that seeking help would negatively affect your career?

- ○ No
- ○ Yes

31. Who discouraged you? (select all that apply)

- ☐ Another medical student
- ☐ Resident/Fellow
- ☐ Professor/Attending Physician
- ☐ School Administrator
- ☐ Healthcare provider (e.g. personal physician)
- ☐ Spouse
- ☐ Other family member

Other (please specify)

Florida Medical Student Wellness Survey

32. Of the following, who was your PRIMARY source of personal support during the past 6 months?

- ○ No One
- ○ Spouse/Romantic partner
- ○ Parent
- ○ Sibling
- ○ Children
- ○ Other Family Member
- ○ Other medical student(s)
- ○ Friend(s) outside of medical school
- ○ 12-Step Sponsor
- ○ Counselor or Therapist
- ○ Peer Advocate
- ○ Professor/Attending Physician
- ○ Medical school Student Affairs officer
- ○ Other

(please specify)

33. In the past 6 months, who did you talk openly with when you had a serious problem, or when you needed someone to be supportive? (select all that apply)

- [ ] No One
- [ ] Spouse/Romantic partner
- [ ] Parent
- [ ] Sibling
- [ ] Children
- [ ] Other Family Member
- [ ] Other medical student(s)
- [ ] Friend(s) outside of medical school
- [ ] 12-Step Sponsor
- [ ] Counselor or Therapist
- [ ] Peer Advocate
- [ ] Professor/Attending Physician
- [ ] Medical school Student Affairs officer
- [ ] Other

Other (please specify)

34. Which over-the-counter medications have you taken in the past 30 days? (select all that apply)

- ☐ Allergy Medications (e.g. Benadryl, Claritin, Sudafed)
- ☐ Cold remedies (e.g. Alka Seltzer, Dristan, Robitussin and Vicks)
- ☐ Diuretics (e.g. Diurex/Pamabrom)
- ☐ Laxatives (e.g. Ex-Lax)
- ☐ Nicotine replacement (e.g. Commit, Nicotrol, Nicoderm)
- ☐ Pain Relievers/fever reducers (e.g. Advil, Alleve, Bayer, Excedrin, Motrin, Tylenol)
- ☐ Sleep Aids (e.g. Unisom, Tylenol PM)
- ☐ Sleep prevention (e.g. No Doz)
- ☐ Weight Loss (e.g. Alli, Dexatrim, Metabolife)
- ☐ I have not taken any over-the-counter medication in the past 30 days
- ☐ Other

(please specify)

Florida Medical Student Wellness Survey

35. Which prescription medications have you taken <u>in the past 30 days</u>? (Select all that apply) Please include ALL medications, regardless of how you obtained them. DO NOT include vitamins or birth control.

- ☐ Anticonvulsants (e.g. Depakote, Lamictal)
- ☐ Antidepressants (e.g. Lexapro, Paxil, Pristiq)
- ☐ Antipsychotics (e.g. Abilify, Seroquel, Zyprexa)
- ☐ Antiretrovials (e.g. Epivir, Isentress, Nevirapine)
- ☐ Barbiturates (e.g. Fioricet)
- ☐ Benzodiazepines (e.g. Ativan, Xanax, Klonopin)
- ☐ Beta Blockers (e.g. Propranolol)
- ☐ Chemotherapy (e.g. Xeloda, Cytoxan, Idamycin, Gleevec)
- ☐ Combination Drugs (e.g. Symbyax)
- ☐ Diuretics (e.g. Lasix)
- ☐ Erectile Dysfunction Drugs (e.g. Viagra, Cialis)
- ☐ Hormone Replacement therapy
- ☐ Muscle Relaxers (e.g. Carisoprodol, Soma)
- ☐ Opioids (e.g. Oxycontin, Vicodin, Methadone, Percocet)
- ☐ Sleep Aids (e.g. Lunesta, Ambien)
- ☐ Stimulants (e.g. Adderall, Concerta, Vyvanse)
- ☐ I have not taken any prescription medications in the past 30 days
- ☐ Other

(please specify)

36. Which other types of medications or supplements have you taken in the past 30 days? (select all that apply) Please include ALL medications and supplements, regardless of how you obtained them. DO NOT include vitamins or birth control.

- [ ] Coffee/espresso or caffeinated soda
- [ ] High-caffeine drinks (e.g. Red Bull, Monster, etc.)
- [ ] Energy shots (e.g. 5 hour energy, Rockstar Energy Shot, NOS PowerShot)
- [ ] Herbal remedies (e.g. Salvia, Valerian, Melatonin, St. Johns wort, etc.)
- [ ] Medical marijuana
- [ ] I haven't used any other medications or supplements in the past 30 days
- [ ] Other

(please specify)

THE FOLLOWING ITEMS ASK ABOUT TOBACCO, ALCOHOL, AND OTHER DRUG USE. PLEASE BE COMPLETELY HONEST WHEN ANSWERING THESE QUESTIONS. NO IDENTIFYING INFORMATION IS BEING COLLECTED ABOUT YOU OR YOUR MEDICAL SCHOOL. ALL RESPONSES ARE COMPLETELY ANONYMOUS AND CANNOT BE LINKED TO YOU IN ANY WAY.

37. Have you ever smoked cigarettes?

- ○ Yes
- ○ No

38. At what age did you smoke your first cigarette?

Age in years

39. Have you ever tried to quit or cut down on your cigarette smoking?

- ○ Yes, both before and during medical school
- ○ Yes, only during medical school
- ○ Yes, only before medical school
- ○ No, never

40. Have you smoked any cigarettes in the past 6 months?

- ○ Yes
- ○ No

41. On the days that you smoked in the past 6 months, about how many cigarettes would you usually smoke in a day?

- Less than 5 cigarettes
- 5 to 14 cigarettes per day
- 15 to 25 cigarettes per day
- 26 to 40 cigarettes per day
- More than 40 cigarettes per day

42. Have you ever smoked electronic or "e-cigarettes"?

- Yes
- No

43. How would you describe your usual pattern of smoking e-cigarettes in the past 6 months?

- I have not used e-cigarettes in the past 6 months
- Every day
- 5 or 6 days a week
- 3 or 4 days a week
- 1 or 2 days a week
- Less than once a week

44. Have you used smokeless tobacco like snuff, chew, dip, or any others more than 5 times in your life?

- Yes
- No

45. How would you describe your usual pattern of using smokeless tobacco in the past 6 months?

- I have not used smokeless tobacco in the past 6 months
- Every day
- 5 or 6 days a week
- 3 or 4 days a week
- 1 or 2 days a week
- Less than once a week

46. What other kinds of tobacco have you used in the past 6 months? (select all that apply)

- [ ] I have not used any other form of tobacco
- [ ] Cigars or Cigarillos
- [ ] Hookah
- [ ] Pipe
- [ ] Other tobacco products (e.g., kreteks, bidis, snus)

47. Have you ever had an alcoholic drink in your life?

- ○ Yes
- ○ No

48. How many standard alcoholic drinks did you have in the past 7 days?

1 standard drink = 1 beer, 1 glass of wine, 1 shot of hard liquor

Total # of standard drinks ____________

49. How would you describe this amount?

- ○ Significantly less than usual
- ○ Slightly less than usual
- ○ About the same as usual
- ○ Slightly more than usual
- ○ Significantly more than usual

50. On the days that you drank in the past 6 months, how much wine, beer, or liquor would you usually have in a day?

1 standard drink = 1 beer, 1 glass of wine, 1 shot of hard liquor

Total # of standard drinks ____________

51. On the day you drank THE MOST in the past 6 months, how much wine, beer, or liquor did you have?

1 standard drink = 1 beer, 1 glass of wine, 1 shot of hard liquor

Total # of standard drinks ____________

52. On how many days in the past 6 months did you drink that much?

Total # of days ____________

53. During medical school, have you had more than 3 drinks (if female) or more than 4 drinks (if male) in one day on an exam day or the day after?

- ◯ Yes, usually
- ◯ Yes, often
- ◯ Yes, once or twice
- ◯ No, never

54. How has your drinking behavior changed since beginning medical school?

- ◯ I began drinking significantly more alcohol
- ◯ I began drinking slightly more alcohol
- ◯ No change
- ◯ I began drinking slightly less alcohol
- ◯ I began drinking significantly less alcohol

55. Have you ever tried to quit or cut down on your drinking?

- ◯ Yes, both before and during medical school
- ◯ Yes, only during medical school
- ◯ Yes, only before medical school
- ◯ No, never

56. In your lifetime, have you ever felt you had a drinking problem?

- ◯ Yes, both before and during medical school
- ◯ Yes, only during medical school
- ◯ Yes, only before medical school
- ◯ No, never

57. Have you ever used marijuana (cannabis, weed, hashish)?

- ◯ Yes, both before and during medical school
- ◯ Yes, only during medical school
- ◯ Yes, only before medical school
- ◯ No, never

58. How has your use of marijuana changed since beginning medical school?

- ◯ I began using significantly more
- ◯ I began using slightly more
- ◯ No change
- ◯ I began using slightly less
- ◯ I began using significantly less

59. On average, how would you describe your usual pattern of marijuana use in the past 6 months?

- ◯ 5-7 days per week
- ◯ 2-4 days per week
- ◯ About 1 day per week
- ◯ 1-3 days per month
- ◯ At least once, but less than 1 day per month
- ◯ I have not used at all in the past 6 months

60. Which of the following substances have you ever used? (Select all that apply)

- ☐ Spice, K2, "bath salts," "herbal incense," or other synthetic drugs
- ☐ Inhalants (nitrous oxide, Dust-Off, paint thinner, glue)
- ☐ NON-PRESCRIPTION stimulants (cocaine, crack, methamphetamine, speed)
- ☐ Ecstasy (X, MDMA)
- ☐ NON-PRESCRIPTION opiates (opium, heroin)
- ☐ Hallucinogens (LSD, PCP, mushrooms)
- ☐ Anabolic steroids
- ☐ None

61. When did you use the substance(s) listed in the previous question?

- ◯ Both before and during medical school
- ◯ Only during medical school
- ◯ Only before medical school

62. In general, how has your overall use of the substance(s) changed since beginning medical school?

- ◯ I began using significantly more
- ◯ I began using slightly more
- ◯ No change
- ◯ I began using slightly less
- ◯ I began using significantly less

63. On average, how would you describe your usual pattern of using the substance(s) noted previously <u>in the past 6 months</u>?

- ◯ 5-7 days per week
- ◯ 2-4 days per week
- ◯ About 1 day per week
- ◯ 1-3 days per month
- ◯ At least once, but less than 1 day per month
- ◯ I have not used at all in the past 6 months

64. Have you ever (even 1 time) used a prescription stimulant like Adderall, Ritalin, Concerta, or Vyvanse, <u>either with or without a prescription</u>?

- ◯ Yes, both before and during medical school
- ◯ Yes, only during medical school
- ◯ Yes, only before medical school
- ◯ No, never

65. How has your use of prescription stimulants changed since beginning medical school?

- ◯ I began using significantly more
- ◯ I began using slightly more
- ◯ No change
- ◯ I began using slightly less
- ◯ I began using significantly less

66. __During medical school__, have you taken prescription stimulants

	Yes	No
to get high?	◯	◯
out of curiosity?	◯	◯
because you were pressured to take them?	◯	◯
to stay awake?	◯	◯
to eat less or lose weight?	◯	◯
to help you study?	◯	◯
to take an exam?	◯	◯
because of competitive nature of medical school?	◯	◯
to relax?	◯	◯
just because?	◯	◯
to treat ADHD symptoms?	◯	◯
because your parents or doctor told you to?	◯	◯
because they seem safer than street drugs?	◯	◯

67. On average, how would you describe your usual pattern of prescription stimulant use __in the past 6 months__?

- ◯ 5-7 days per week
- ◯ 2-4 days per week
- ◯ About 1 day per week
- ◯ 1-3 days per month
- ◯ At least once, but less than 1 day per month
- ◯ I have not used at all in the past 6 months

68. Were the prescription stimulants you used prescribed for you?

- ◯ Yes, every time
- ◯ Yes, most of the time
- ◯ Sometimes yes, sometimes no
- ◯ No, never

69. Have you ever (even 1 time) used a prescription opiate/opioid like Vicodin, Oxycontin, Percocet, or Methadone either with or without a prescription?

- ◯ Yes, both before and during medical school
- ◯ Yes, only during medical school
- ◯ Yes, only before medical school
- ◯ No, never

70. How has your use of prescription opiates/opioids changed since beginning medical school?

- ◯ I began using significantly more
- ◯ I began using slightly more
- ◯ No change
- ◯ I began using slightly less
- ◯ I began using significantly less

71. During medical school, have you taken prescription opiates/opioids

	Yes	No
to get high?	◯	◯
out of curiosity?	◯	◯
because you were pressured to take them?	◯	◯
to function?	◯	◯
to relax, calm down, or relieve stress?	◯	◯
to sleep?	◯	◯
to relieve pain?	◯	◯
just because?	◯	◯
because your parents or doctor told you to?	◯	◯
because they seem safer than street drugs?	◯	◯

72. On average, how would you describe your usual pattern of prescription opiate/opioid use in the past 6 months?

- ◯ 5-7 days per week
- ◯ 2-4 days per week
- ◯ About 1 day per week
- ◯ 1-3 days per month
- ◯ At least once, but less than 1 day per month
- ◯ I have not used at all in the past 6 months

73. Were the prescription opiates/opioids you used prescribed for you?

- ◯ Yes, every time
- ◯ Yes, most of the time
- ◯ Sometimes yes, sometimes no
- ◯ No, never

74. Have you ever tried to quit or cut down on use of ANY illicit or prescription drugs?

- ◯ Yes, both before and during medical school
- ◯ Yes, only during medical school
- ◯ Yes, only before medical school
- ◯ No, never

75. In your lifetime, have you ever felt you had a problem with ANY drug use?

- ◯ Yes, both before and during medical school
- ◯ Yes, only during medical school
- ◯ Yes, only before medical school
- ◯ No, never

THE NEXT ITEMS ASK ABOUT GENERAL LIFESTYLE HABITS AND OVERALL WELL-BEING. PLEASE ANSWER HONESTLY. YOUR RESPONSES WILL REMAIN ANONYMOUS.

76. How would you rate your level of happiness since beginning medical school?

- ◯ Extremely happy
- ◯ Moderately happy
- ◯ Somewhat happy
- ◯ Somewhat unhappy
- ◯ Moderately unhappy
- ◯ Extremely unhappy

77. How has your overall level of happiness changed since beginning medical school?

- ◯ Significantly happier
- ◯ Moderately happier
- ◯ Slightly happier
- ◯ No change
- ◯ Slightly less happy
- ◯ Moderately less happy
- ◯ Significantly less happy

78. Have you ever felt sad or depressed most days for a period of 2 weeks or more, or have you ever been told by a health professional that you had depression?

- ◯ Yes, and the symptoms began or worsened since starting medical school
- ◯ Yes, but the symptoms have improved since starting medical school
- ◯ Yes, but only before medical school
- ◯ No, never

79. Some people become extremely anxious when performing specific tasks (e.g., eating, public speaking) in front of other people because they fear they will seriously embarrass or humiliate themselves. They may try to avoid social interactions or may have panic attacks when forced to participate.

Have you ever experienced clinically-significant problems with this, or have you ever been told by a health professional that you had social anxiety or social phobia?

- ◯ Yes, and the symptoms began or worsened since starting medical school
- ◯ Yes, but the symptoms have improved since starting medical school
- ◯ Yes, but only before medical school
- ◯ No, never

80. Some people tend to be “worriers,” spending a lot of time thinking about things that have gone or could go wrong. They might have trouble falling asleep or have difficulty concentrating, and often describe feeling “on edge” or anxious. They may have “anxiety attacks.”

Have you ever experienced a period of 6 months or more when you experienced clinically-significant problems with this, or have you ever been told by a health professional that you had generalized anxiety disorder?

- ◯ Yes, and the symptoms began or worsened since starting medical school
- ◯ Yes, but the symptoms have improved since starting medical school
- ◯ Yes, but only before medical school
- ◯ No, never

81. Some people experience intrusive thoughts, images, or sounds that get "stuck in their head" or come back repeatedly. As a result, they avoid certain places or things, or may engage in rituals or routines to feel better, like frequent handwashing, counting, checking, ordering or arranging, redoing, etc. These thoughts and behaviors take up a considerable amount of time in their day.

Have you ever experienced clinically-significant problems with this, or have you ever been told by a health professional that you had obsessive-compulsive disorder?

- ◯ Yes, and the symptoms began or worsened since starting medical school
- ◯ Yes, but the symptoms have improved since starting medical school
- ◯ Yes, but only before medical school
- ◯ No, never

82. Some people become extremely preoccupied by food and weight concerns. They may have a distorted body image. Some will "binge eat" extremely large amounts of food in a short period of time. Some may severely restrict their eating and/or engage in vomiting, excessive exercise, or use of laxatives to control their weight.

Have you ever experienced clinically-significant problems with this, or have you ever been told by a health professional that you had an eating disorder?

- ◯ Yes, and the symptoms began or worsened since starting medical school
- ◯ Yes, but the symptoms have improved since starting medical school
- ◯ Yes, but only before medical school
- ◯ No, never

83. Some people have been very easily distracted since childhood and have great difficulty staying organized.They have a lot of trouble staying focused and in some cases, they have difficulty "sitting still" or feel hyperactive.

Have you experienced clinically-significant problems with this, or have you ever been told by a health professional that you had ADD or ADHD?

- ◯ Yes, and the symptoms began or worsened since starting medical school
- ◯ Yes, but the symptoms have improved since starting medical school
- ◯ Yes, but only before medical school
- ◯ No, never

84. Some people have periods of a month or more with difficulty falling asleep, waking frequently during the night, waking up early in the morning, or having severe nightmares.

Have you ever experienced clinically-significant problems with this, or have you ever been told by a health professional that you had a sleep disorder?

- ◯ Yes, and the symptoms began or worsened since starting medical school
- ◯ Yes, but the symptoms have improved since starting medical school
- ◯ Yes, but only before medical school
- ◯ No, never

85. Many people bet or gamble. Some people become preoccupied with going to casinos, online gambling, playing the lottery, or betting on sports. Their gambling may cause problems at home, work, school, in relationships, or with finances.

Have you ever experienced clinically-significant problems related to gambling, or have you ever been told by a health professional that you had a gambling problem?

- ◯ Yes, and the symptoms began or worsened since starting medical school
- ◯ Yes, but the symptoms have improved since starting medical school
- ◯ Yes, but only before medical school
- ◯ No, never

86. Have you ever been sexually active?

- ◯ Yes, both before and during medical school
- ◯ Yes, only during medical school
- ◯ Yes, only before medical school
- ◯ No, never

87. How have your sexual habits changed since beginning medical school?

- ◯ Significantly more sexual activity
- ◯ Slightly more sexual activity
- ◯ No change
- ◯ Slightly less sexual activity
- ◯ Significantly less sexual activity

88. <u>In the last 4 months</u>, how many different sex partners have you had? (include oral, anal, and vaginal sex partners)

# of partners in past 4 months __________

89. How would you currently describe your spirituality/spiritual practice? (select all that apply)

- ☐ None
- ☐ Believe in power greater than self
- ☐ Describe self as "spiritual"
- ☐ Practice spirituality informally (such as reading spiritual material, prayer, meditation)
- ☐ Regularly attend a church or religious group or place of worship

Florida Medical Student Wellness Survey

THANK YOU FOR YOUR EFFORT SO FAR. YOU ARE ALMOST FINISHED! THE NEXT QUESTIONS WILL BE MOST HELPFUL TO US IN DETERMINING WAYS TO IMPROVE THE MEDICAL SCHOOL EXPERIENCE FOR STUDENTS. PLEASE TAKE A MOMENT TO SHARE YOUR IDEAS.

90. Have you recently questioned whether you really want to be a doctor?

- ◯ No
- ◯ Yes

91. Why?

***92. What do you consider to be the greatest stressor(s) facing medical students?**

***93. What could medical schools do to improve medical student health and wellness?**

***94. If you were to design a student wellness program for your medical school, what would it include and not include?**

Please do not name your medical school in your response.

THANK YOU FOR YOUR PARTICIPATION IN THIS SURVEY. THESE FINAL 6 QUESTIONS WILL BE USED TO ASSIST WITH DATA ANALYSIS. WE GREATLY APPRECIATE YOUR RESPONSES.

95. Who do you currently live with? (select all that apply)

- ☐ I live alone
- ☐ My spouse or significant other
- ☐ My child(ren)
- ☐ My parent(s)
- ☐ My sibling(s)
- ☐ Friend(s)
- ☐ Other relative(s)
- ☐ Roommate(s) not otherwise specified above

Florida Medical Student Wellness Survey

96. In what year of medical school are you currently?

- 1st year
- 2nd year
- 3rd year
- 4th year
- 5th year or higher

97. What gender do you consider yourself to be?

- Male
- Female

98. How honest were you in completing this survey?

- Not at all honest
- Somewhat honest
- Mostly honest
- Completely honest
